# Supplementary material for: Implications of Senescent Cell Burden and NRF2 Pathway in Uremic Calcification: A Translational Study
Source: Cells. 2023 Feb 17;12(4):643. doi: 10.3390/cells12040643 (PMC9954542; doi:10.3390/cells12040643)
Supplement: Supplementary file 1 [file cells-12-00643-s001.zip › cells-2184179-supplementary.pdf]

Supplementary Material

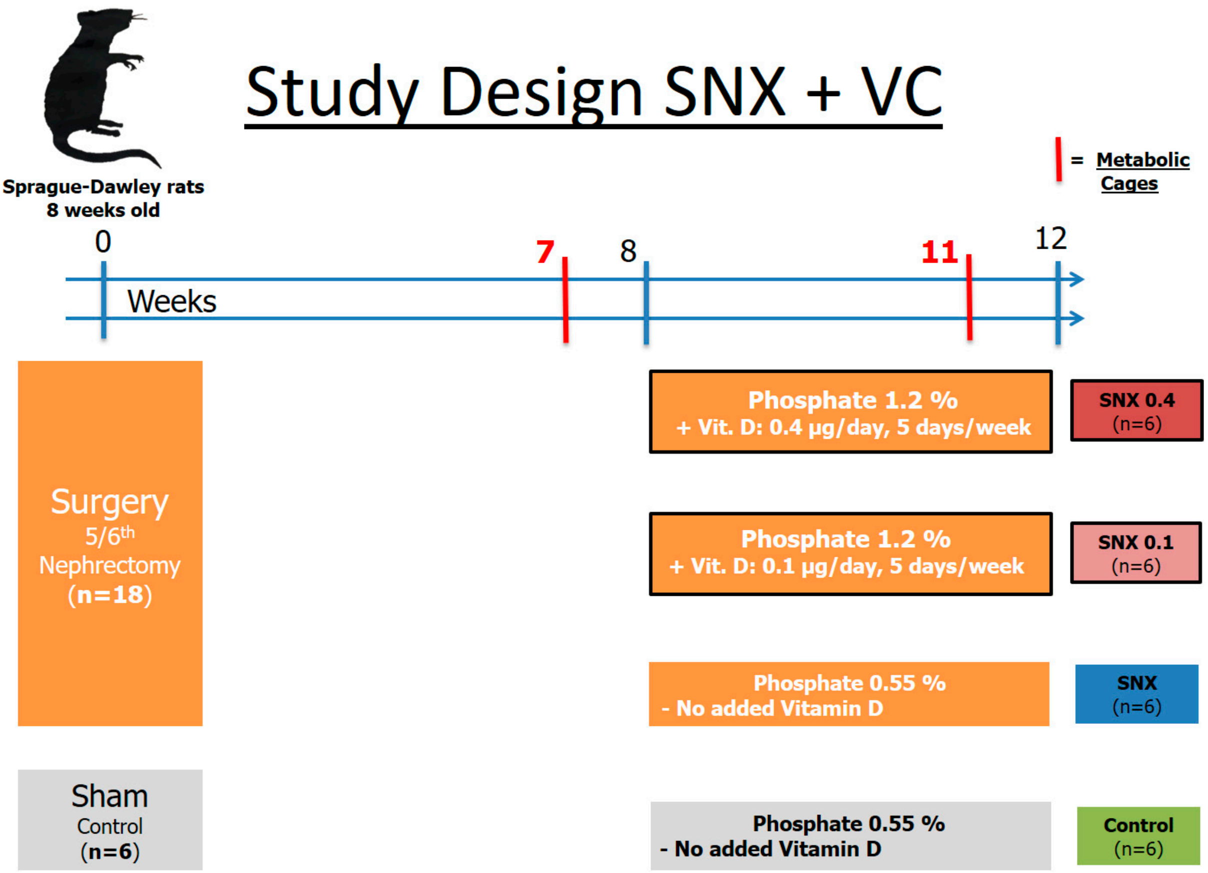

Supplementary Figure S1 – In vivo experiment study design.

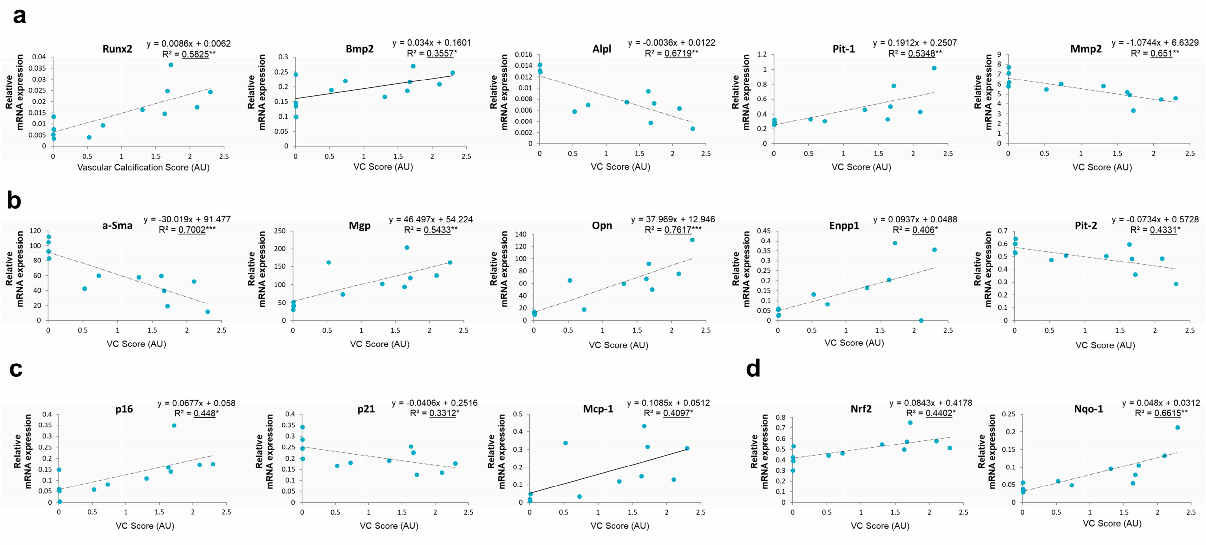

Supplementary Figure S2 – Correlation of mRNA expression in thoracic aorta with vascular calcification score (VC score).

a) mRNA expression of genes promoting vascular calcification correlated with vascular calcification score in thoracic aorta of SNX 0.1 and SNX 0.4 rats.
b) mRNA expression of alpha-SMA and genes inhibiting vascular calcification correlated with VC score in thoracic aorta of SNX 0.1 and SNX 0.4 rats.
c) mRNA expression of $p16^{Ink4a}$, $p21^{Cip1}$ and Mcp-1 correlated with VC score in thoracic aorta of SNX 0.1 and SNX 0.4 rats.
d) Nrf2 and Nqo1 mRNA expression correlated with VC score.***: $p < 0.001$, **: $p < 0.01$, *: $p < 0.05$.

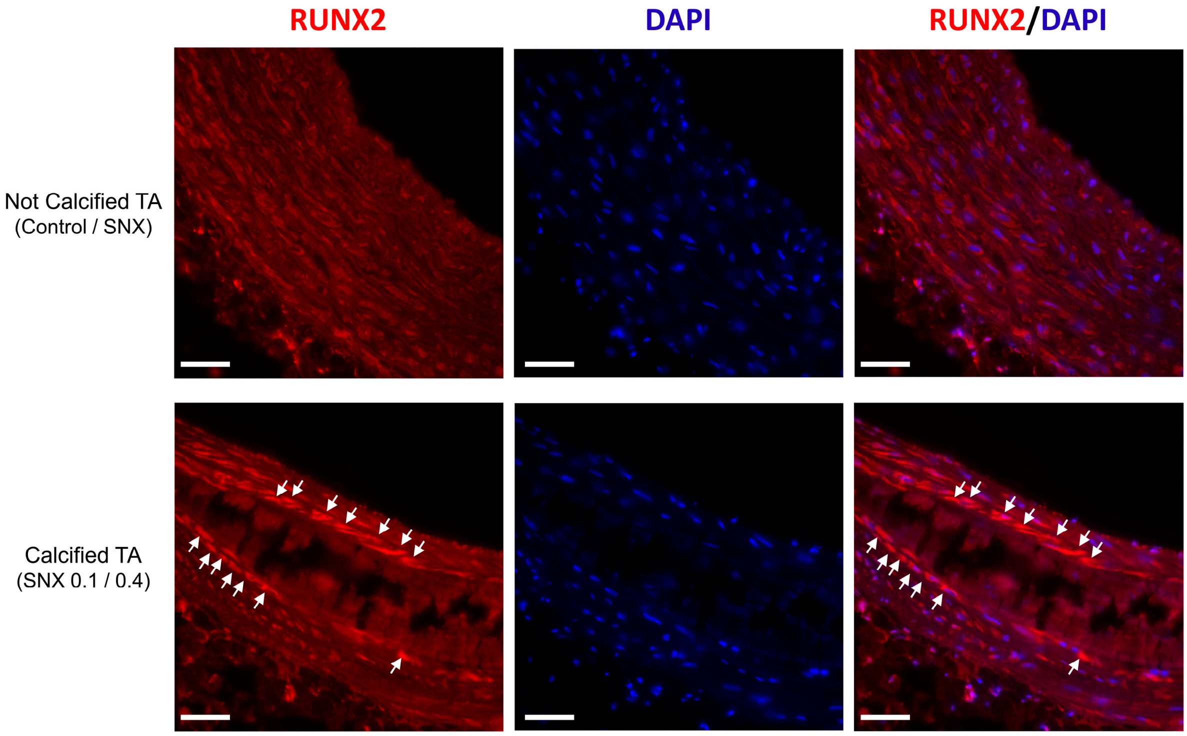

Supplementary Figure S3 – Immunofluorescence of Runx2 in calcified versus non calcified thoracic aortas (TA).

RUNX2 protein expression was observed close to the calcified area in the media layer of TA. White arrows indicate RUNX2-positive cells. Scale bar represent 50 µm.

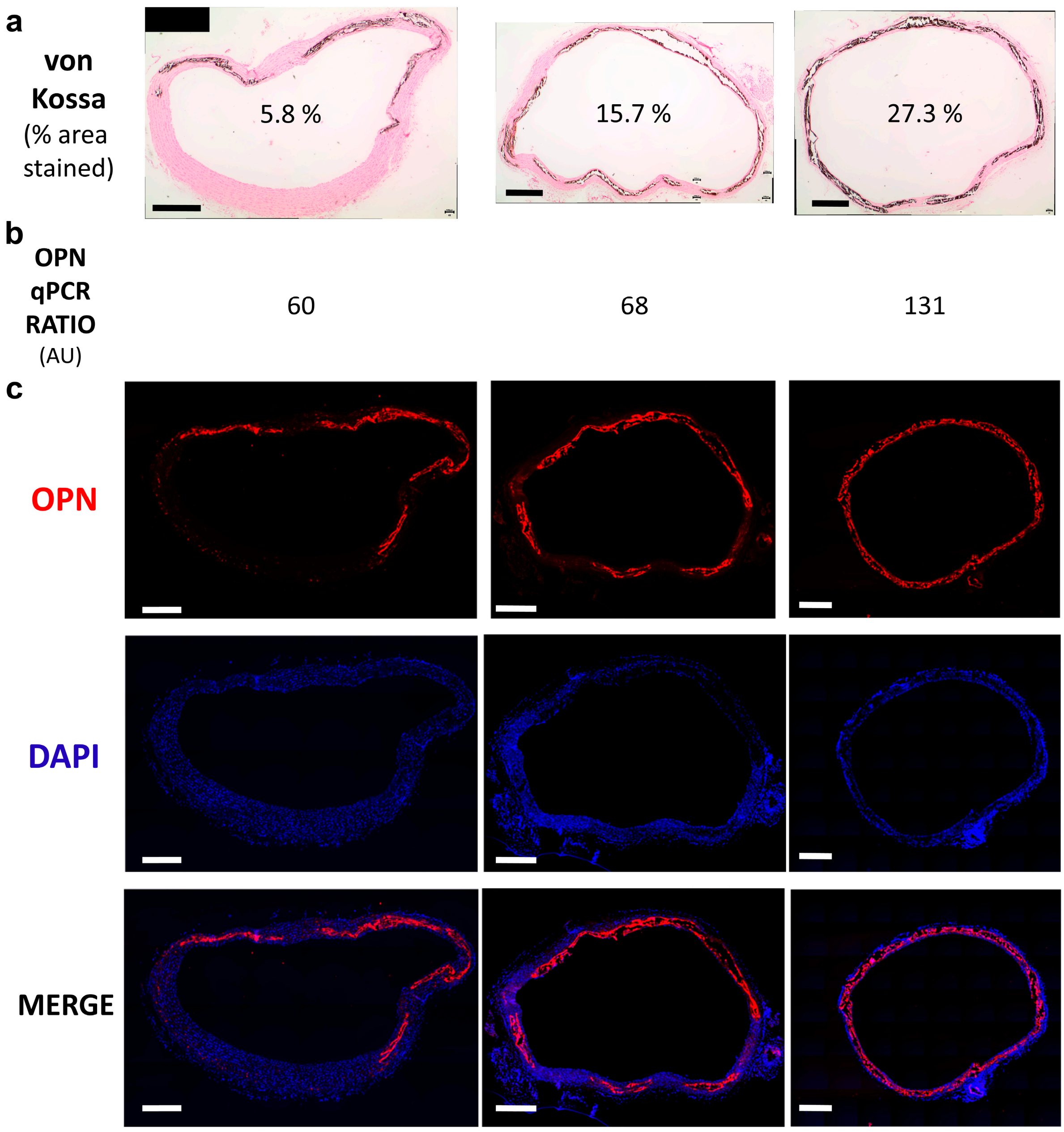

Supplementary Figure S4 – Osteopontin protein and mRNA expression in thoracic aorta from three uremic rats presenting gradual increases of vascular calcification.

a) Representative images of von Kossa staining in thoracic aortas presenting gradual increases in calcification (quantification of von Kossa staining is indicated in the centre of each image).
b) qPCR ratio measured for the respective samples.
c) OPN IF labelling in the same Tas that present increases in calcification.

Scale bars correspond to 500 µm.

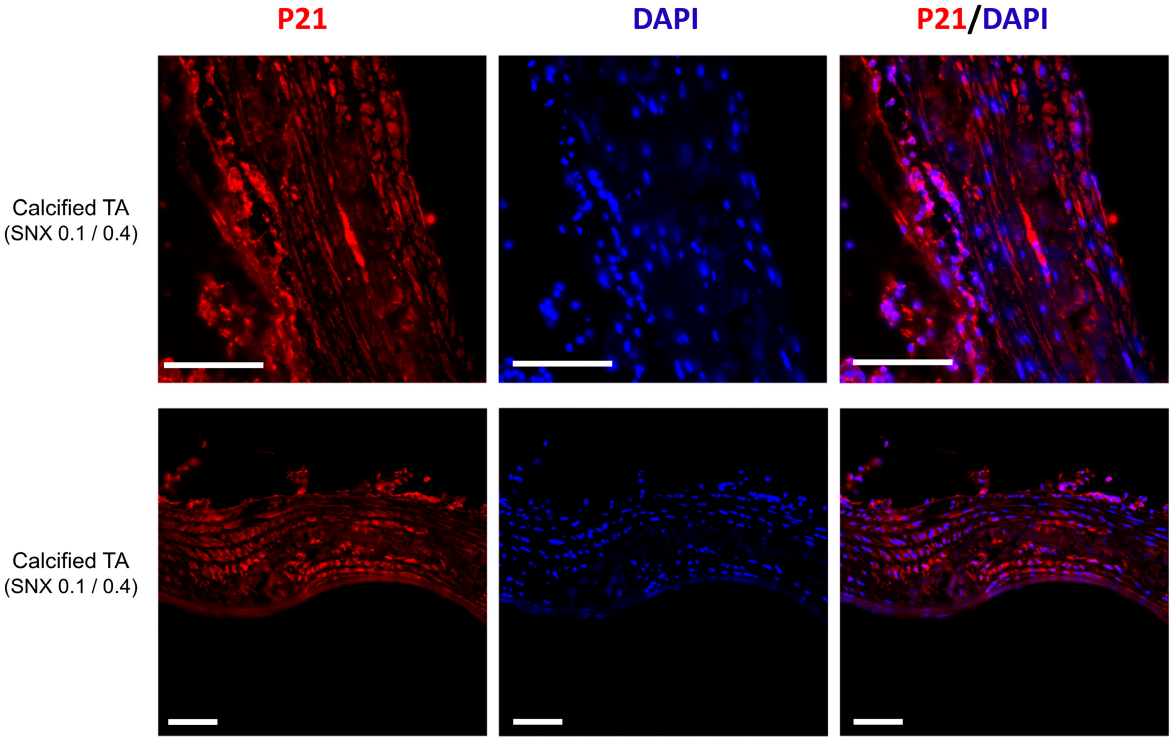

Supplementary Figure S5 – Immunofluorescence for p21^{Cip1} protein expression in calcified thoracic aortas (TA).

P21^{Cip1} labelling was observed close to calcium deposits.

Scale bars represent 100 µm.

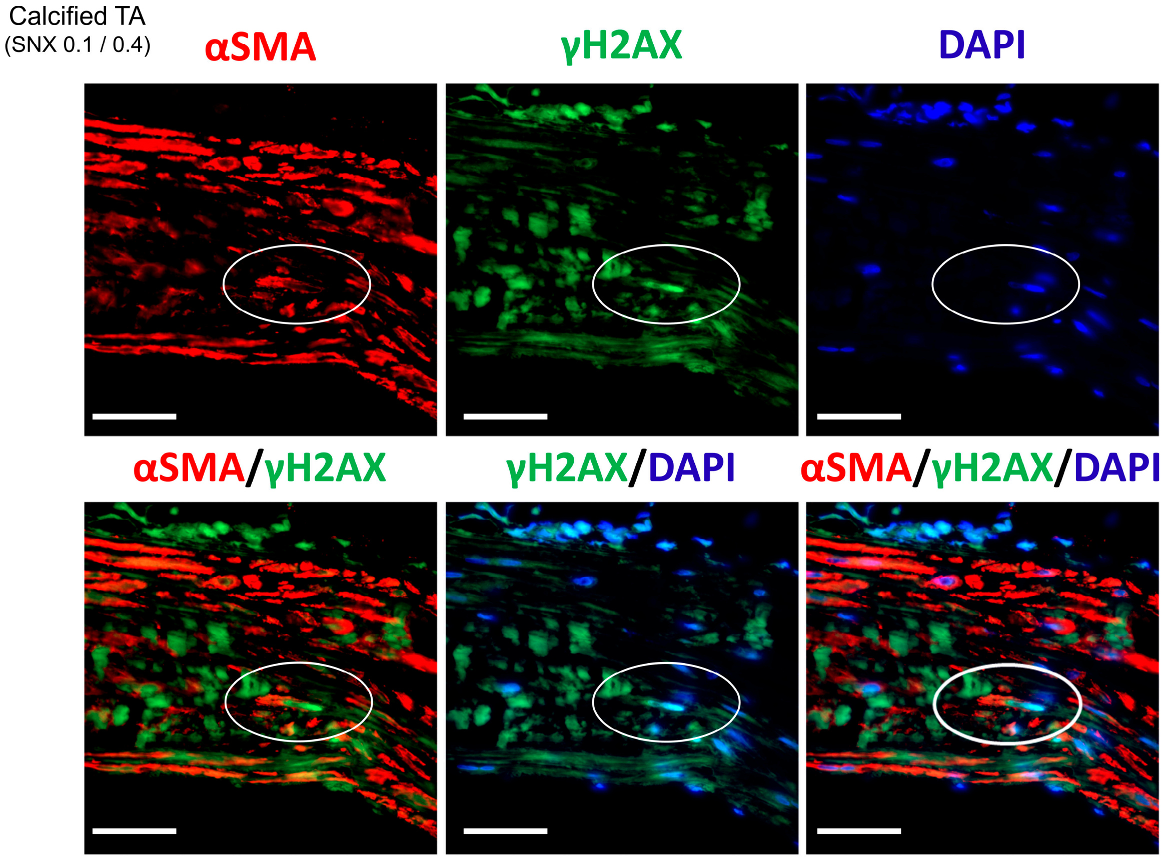

Supplementary Figure S6 – Immunofluorescence of αSMA and γH2AX in calcified thoracic aorta (TA) of uremic rats.
Cells co-labelled with αSMA and γH2AX can be observed close to calcium deposits.
Scale bars represent 50 µm.

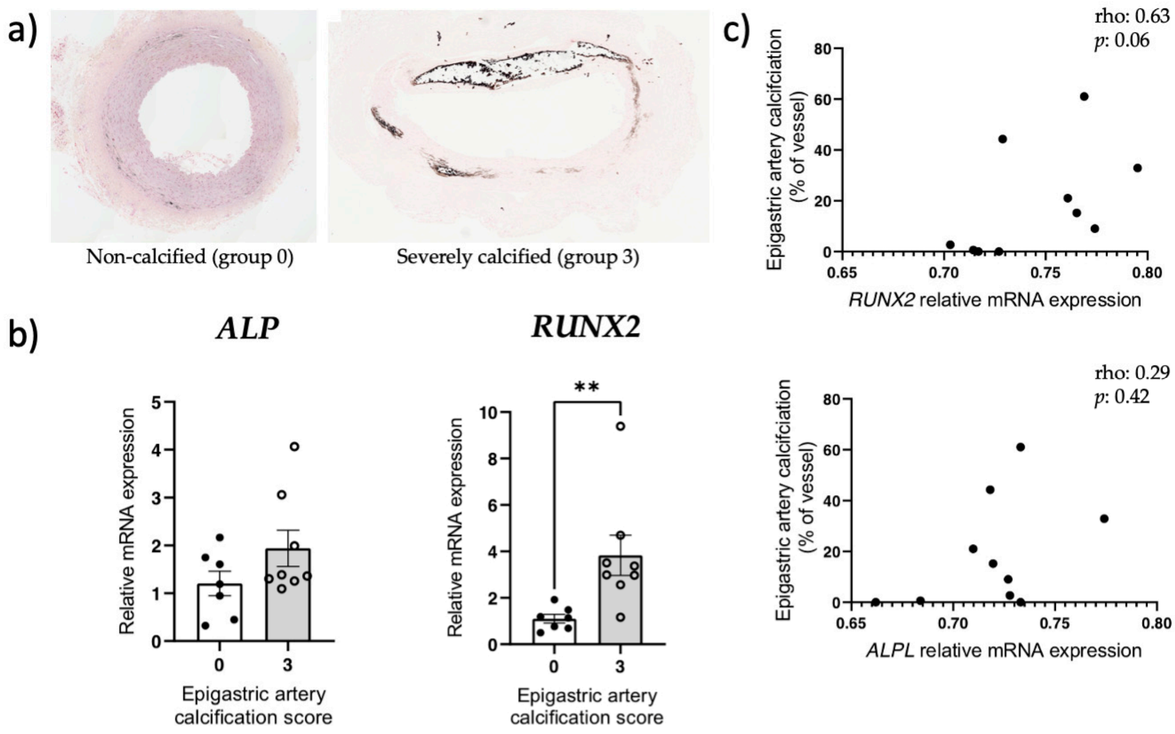

Supplementary Figure S7 – Severely calcified epigastric arteries have increased osteogenic gene expression compared to non-calcified vessels.

a) Representative images of non-calcified (left) and severely calcified (right) epigastric artery segments. Scoring was assessed by a trained pathologist using von Kossa staining.

b) mRNA expression analysis (qPCR) of osteogenic markers *ALPL* and *RUNX2* between non-calcified and severely calcified vessels isolated from kidney failure patients at LD-Ktx. Relative gene expression normalised to non-calcified group. 0= non-calcified epigastric artery, 3= severely calcified epigastric artery.

c) Correlations between *RUNX2* and *ALPL* gene expression and epigastric artery calcification (% of vessel).

Statistics: Differences between groups were assessed using non-parametric Mann–Whitney U test. Spearman rank correlation coefficient is given for non-parametric data. Normality was assessed with Shapiro-Wilk test. P-values <0.05 were deemed statistically significant. ** indicates p<0.01.

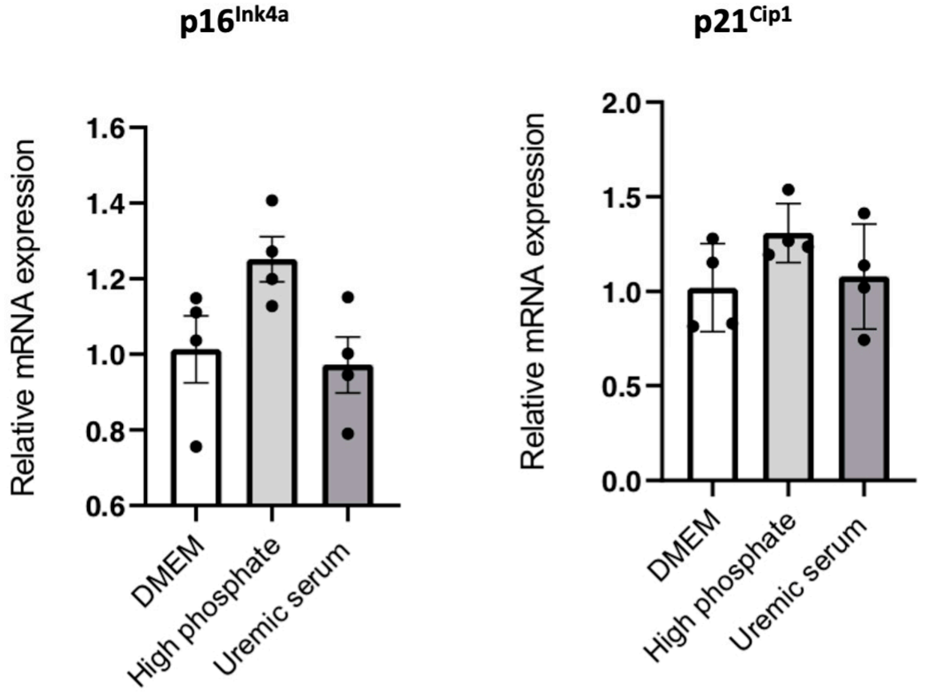

Supplementary Figure S8 – Uremic serum-induced vascular smooth muscle cell calcification is not associated with senescence.

Aortic VSMCs were incubated with pooled uremic serum from severely calcified kidney failure patients (n=8) for 7 days to induce calcification in vitro. Treatment conditions: control DMEM media (DMEM), 2.5mM phosphate media (High phosphate), or 10% pooled uremic serum + high phosphate media (Uremic serum). Relative mRNA expression of $p16^{Ink4a}$ and $p21^{Cip1}$ was normalised to DMEM control group.

	r	P-value
**Correlation of different VC assessment assays with Pulse Pressure (mmHg)**		
von Kossa of AA (% of media area)	0.7819	**0.0027**
Ca content of TA (nmol/mg dry ring)	0.7667	**0.0036**
von Kossa of TA (% of media area)	0.738	**0.0061**
**Correlation of Scores of VC with Pulse Pressure (mmHg)**		
VC score (AU) = $\frac{1}{2}\left(\frac{\text{stained\_AA}}{\text{Mean (stained\_AA (SNX 0.1/0.4))}}+\frac{1}{2}\left(\frac{\text{stained\_TA}}{\text{Mean (stained\_TA (SNX 0.1/0.4))}}+\frac{\text{Ca\_TA}}{\text{Mean (Ca\_TA (SNX 0.1/0.4))}}\right)\right)$	<u>0.7889</u>	**<u>0.0023</u>**

Supplementary Table S1

Table S1 – Correlations between Pulse Pressure and Vascular Calcification assessments in SNX 0.1 and 0.4 groups.

Vascular calcification is characterised by multifocal calcium deposition in the media layer of arteries. Von Kossa staining can specifically assess medial calcification in a small tissue sample, while Ca content is representative of a bigger tissue sample but less specific to medial localisation. The three measurements of vascular calcification (von Kossa staining in aortic arch and thoracic aorta, and Ca content in thoracic aorta) were strongly correlated with pulse pressure (r > 0.7). This is consistent with the fact that vascular calcification is expected to induce arterial stiffness and increase pulse pressure. To mitigate technical limitations and obtain a single VC estimate, we calculated a VC score that displayed an even stronger correlation with pulse pressure. Pearson's correlation coefficients (r) and p-value are given.

RAT Primers

Target	Forward Primers (5'-3')	Reverse Primers (5'-3')	Gene Name and Aliases
**a-Kl**	AGGCACTGGCGACTATAA	GTACTCCCACAGTGTAGAAAC	alpha-Klotho
**a-Sma**	CTTTCATTGGAATGGAGTCG	GACGTTGTTAGCATAGAGGTC	alpha-Smooth Muscle Actin / actin alpha 2 (Acta2)
**Alpl**	ATTTGGAAGAGCTTCAAACCTAGA	CGGGCTCAAAGAGACCTAA	Alkaline Phosphatase, Biomineralization Associated
**B-act**	GATCAAGATCATTGCTCCTCCTG	AGGGTGTAAAACGCAGCTCA	Actin Beta
**Bmp2**	AGGTAAGACCACTGCTAGTGA	GCTGTTTGTGTTTGGCTTG	Bone Morphogenic Protein 2 (Actb)
**Cat**	CACTTTGACAGAGAGCGG	CCTTGGAGTATCTGGTAATATCG	Catalase
**Enpp1**	ACTTTGATTATGATGGACGGT	AGTGAGTGGGAATCAGATTT	Ectonucleotide Pyrophosphatase/Phosphodiesterase 1
**Gpx4**	TGCCTGGATAAGTACAGGG	ATGCAGATCGACTAGCTGA	Glutathione Peroxidase 4
**Gusb**	GCCTGTCTCTTCTCTGAAAC	CCATGTCTGCGTCATATCT	Glucuronidase Beta
**Hprt1**	TCCTCCTCAGACCGCTTTTC	ATCACTAATCACGACGCTGGG	Hypoxanthine Phosphoribosyltransferase 1
**Il-1b**	GGGCCTCAAGGGGAAGAATC	GTTTGGGATCCACACTCTCCA	Interleukin 1 Beta
**Il-6**	CTCTCCGCAAGAGACTTCCAG	TCTGACAGTGCATCATCGCT	Interleukin 6
**Mcp-1**	TGTCTCAGCCAGATGCAGTT	CAGCCGACTCATTGGGATCA	Monocyte Chemoattractant Protein-1 / C-C Motif Chemokine Ligand 2 (CCL2)
**Mgp**	CTCACGAAAGCATGGAATC	GACTCTTTCCTGGGCTTTAG	Matrix Gla Protein
**Mmp2**	CCAGCACTTTGGAAAGAGGATA	TCCAGTTAAAGGCAGCGTCTA	Matrix Metallopeptidase 2
**Msx2**	CTTTCTCTCTGTGTGTGTGTACTTA	TTTCCACCAAGCCCAAC	Msh Homeobox 2
**Nqo1**	CCTTTCTGTGGGCCATC	AAAGAAACCTGGGTCTAACTAC	NAD(P)H Quinone Dehydrogenase 1
**Nrf2**	AGGAGAGGGAAGAATAAAGTTG	ATGGAGGTTTCTGTCGTT	Nuclear factor erythroid 2-related factor 2 / NFE2 Like BZIP Transcription Factor 2 (NFE2L2)
**Opn**	CTCTGAAGAAACGGATGACT	GAATCCTCGCTCTCTGC	Osteopontin / Secreted Phosphoprotein 1 (SPP1)
**p16**	TGCGGTATTTGCGGTATC	CGTGCTTGAGCAGAAGTT	Cyclin Dependent Kinase Inhibitor 2A (CDKN2A)
**p21**	GACCAGCCTAACAGATTTCTAT	TTAAGACACACTGAATGAAGGCTA	Cyclin Dependent Kinase Inhibitor 1A (CDKN1A)
**p53**	TCCTTACCATCATCACGCT	GACAGGCACAAACACGA	Tumor Protein P53 (TP53)
**Pit-1**	GGGAAGAAGAGTTATCCAGACTA	CCAATGTTTGATGCGACG	Solute Carrier Family 20 Member 1 (SLC20A1)
**Pit-2**	TTGTTTCTTCCAGTCGGTAAAT	TGCAAGTGTCAGAGAAAGC	Solute Carrier Family 20 Member 2 (SLC20A2)
**Runx2**	TGATGACTCTAAACCTAGTTTGTTC	CACCTACTCTCATACTGGGAT	RUNX Family Transcription Factor 2
**Sod1**	CAATGTGTCCATTGAAGATCG	TTTGCCCAAGTCATCTTGTT	Superoxide Dismutase 1
**Tgf-b1**	CTGGAAAGGGCTCAACACCT	CTTCTCTGTGGAGCTGAAGCA	Transforming Growth Factor Beta 1

HUMAN Primers

Target	Forward Primers (5'-3')	Reverse Primers (5'-3')	Gene Name and Aliases
**Alpl**	GGGACTGGTACTCAGACAACG	GTAGGCGATGTCCTTACAGCC	Alkaline Phosphatase, Biomineralization Associated
**Cat**	CGGAACAACAGCCTTCTGCC	GTGGCCGGCAATGTTCTCAC	Catalase
**Gapdh**	GAGTCAACGGATTTGGTCGT	GACAAGCTTCCCGTTCTCAG	Glyceraldehyde-3-phosphate Dehydrogenase
**Msx2**	TGCAGAGCGTGCAGAGTTC	GGCAGCATAGGTTTTGCAGC	Msh Homeobox 2
**Nqo1**	AGGCTGGTTTGAGCGAGTGT	ATGTCCCCGTGGATCCCTTG	NAD(P)H Quinone Dehydrogenase 1
**Nrf2**	TCTGACTCCGGCATTTCACT	GGCACTGTCTAGCTCTTCCA	Nuclear factor erythroid 2-related factor 2 / NFE2 Like BZIP Transcription Factor 2 (NFE2L2)
**p16**	CTGCCCAACGCACCGAATAG	GAGGCTCGCAAGAAATGCCC	Cyclin Dependent Kinase Inhibitor 2A (CDKN2A)
**p21**	AGCTGCCGAAGTCAGTTCCT	GGTTCTGACGGACATCCCCA	Cyclin Dependent Kinase Inhibitor 1A (CDKN1A)
**Runx2**	TTCACCTTGACCATAACCGTC	GGCGGTCAGAGAACAAACTAG	RUNX Family Transcription Factor 2
**Sod1**	CGAGTTATGGCGACGAAGGC	GGACCTGCACTGGTACAGCC	Superoxide Dismutase 1

Supplementary Table S2. Rat and human primer sequences designed for qPCR experiments.

Supplementary Materials and Methods

Animal model of uremic vascular calcification

Animals, tissue sampling and blood parameters. Twenty-four male Sprague Dawley (OFA) rats (Charles River Laboratories, France) were housed in specific facilities (permit D3417225) with a 12h light/dark cycle in temperature-controlled conditions (22 ± 1˚C) and fed normal rat chow (A04, SAFE) diet and tap water ad libitum. Eighteen eight week-old rats underwent 5/6th nephrectomy (SNX) in a one-step procedure and six rats underwent a sham operation (control group).[28] Briefly, under isoflurane (2% in O_2) volatile anaesthesia and buprenorphine analgesia (Bupaq, 0.1 mg/kg, subcutaneous injection), a ventral laparotomy was performed to expose the kidneys. The right kidney was excised, and two of the three branches of the left renal artery were ligated. Six sham-operated rats followed laparotomy and manipulation of the two kidneys without renal mass reduction. Analgesia was maintained for 48h post-surgery (buprenorphine 0.1 mg/kg/12h, subcutaneous injection). Rats were left eight weeks without manipulation for the establishment of CKD pathophysiology (Supplemental Figure S1). Twelve SNX rats (≈500g weight) then initiated a 4-week VC-diet – standard diet supplemented with high phosphate (1.2 % P, 1.04% Ca, SAFE) and 1-hydroxy-vitamin D in food pellet (Alfacalcidol, LEO pharma) at a dose of 0.1 µg/day/rat (SNX 0.1 group, n=6) or 0.4 µg/day/rat (SNX 0.4 group, n=6), while sham-operated rats (Control, n=6), and part of the SNX group (SNX, n=6) were maintained on a standard diet (A04 (0.55% P, 0.73% Ca), SAFE). After four weeks, rats were put in metabolic cages for three days to obtain urine production. Arterial pressure was then measured under isoflurane anaesthesia (2% in O_2) after catheterisation of the right carotid. Euthanasia was performed with pentobarbital (200 mg/kg, intravenous injection) before blood and organs (kidney, heart, aortic arch, thoracic aorta) were harvested. Creatinine, urea, total protein, phosphate, and calcium concentrations were measured in plasma and urine on a COBAS automated analyser (Roche Diagnostics, France).

We considered that an increase in pulse pressure in calcified groups was necessary to validate our model. To detect a 50% increase in pulse pressure in calcified SNX (SNX 0.1 and 0.4) and considering a pulse pressure of 60 mmHg in SNX group, standard deviations of 10 mmHg in SNX and 20 mmHg in calcified SNX, a risk alpha = 5% and a power beta = 90 %, it was necessary to include 6 animal in each group. (https://www.bu.edu/researchsupport/compliance/animal-care/working-with-animals/research/sample-sizecalculations-iacuc/)

All animal experiments were performed according to the European Parliament Directive 2010/63/EU (N° CEEA-00322.03) and approved by the local ethics committee for animal experimentation of Languedoc-Roussillon (CEEA-LR, n°036, #18348). This study is reported in accordance with ARRIVE guidelines when applicable.[36]

Histology. 5 µm-sections of kidney or heart (Formalin Fixed and Paraffin Embedded, FFPE) were cut with a microtome (HistoCore Multicut, Leica), stained with 0.1% picrosirius red, mounted in Eukitt medium (Sigma-Aldrich), and photographed (NIKON Eclipse TE300 microscope and Sony-Exmor CMOS P6 digital camera) for determination of fibrosis. Red positive staining was quantified using Image J software.

Thoracic aorta FFPE sections were stained by von Kossa and imaged (see above). Black positive staining was quantified (Image J) in the medial layer and expressed as % of media area. Calcium content of thoracic aorta was determined by the o-cresolphthalein complexone method (Calcium Colorimetric Assay Kit, Biovision). VC was also assessed in aortic arch by von Kossa staining. After staining, aortic arches were cut longitudinally, fixed with needles to expose the inner side, and photographed (Tough TG-6, Olympus) before positive staining was

measured (Image J). To obtain an integrated estimate of vascular calcification, a score accounting for two tissues (aortic arch (AA) and thoracic aorta (TA)) and two methods (von Kossa and Ca content) was established (Supplementary table S1) according to the following formula (Figure 1d):

$$\text{VC score}= \frac{1}{2}\left(\frac{\text{stained\_AA}}{\text{Mean (stained\_AA (SNX 0.1/0.4))}} + \frac{1}{2}\left(\frac{\text{stained\_TA}}{\text{Mean (stained\_TA (SNX 0.1/0.4))}} + \frac{\text{Ca\_TA}}{\text{Mean (Ca\_TA (SNX 0.1/0.4))}}\right)\right)$$

VC score, only calculated for SNX 0.1 and SNX 0.4, was used to test for correlation with other biological parameters.

For immunohistochemistry experiments, 5 µm-sections of thoracic aorta (FFPE) were incubated with primary antibodies and revelation was performed with the Vectastain ABC kit and ImmPACT AEC 130 (Vector Labs) before images were acquired (see above). For Immunofluorescence, 5 µm-sections of thoracic aorta (FFPE) were incubated with a primary antibody and appropriate secondary antibody. Fluorescence was observed with Zeiss Axioimager Z Apotome using Montpellier MRI imaging platform and analysed with Omero software. We used anti-p16 (SAB5300498, Sigma), anti-p21 (ab80633, Abcam), anti-NRF2 (ab137550, Abcam), anti-γH2AX (9718, Cell Signalling), anti-OPN (18628, IBL international), anti-Runx2 (ab76956, Abcam), and anti-αSMA (ab7817, Abcam) primary antibodies. Anti-rabbit IgG (4412 and 8889, Cell Signalling) or anti-mouse IgG (4408 and 8890, Cell Signaling) secondary antibodies were used.

mRNA expression. Total RNA extraction was performed on thoracic aorta using TRI reagent (T9424, Sigma-Aldrich) and FastPrep-24 instrument (MP Biomedicals). RNA concentrations, quality, and purity were evaluated using a Nanodrop One (ThermoFisher Scientific) and agarose gel electrophoresis. First strand cDNA was synthesized from 500 ng RNA using SuperScript III Reverse Transcriptase (Invitrogen) and oligo(dT)$_{20}$ primers. Quantitative PCR (qPCR) was performed using 2.5 ng cDNA, LightCycler 480 SYBR Green I Master mix (Roche), and 500 nM specific primers (Supplementary Table S2) in 10 µl reaction volume on a LightCycler 96 system (Roche). Data were analysed with LightCycler 96 software 1.1 and mRNA expression quantified as Relative Ratio using three housekeeping genes: β-act, Gusb and Hprt1.

Observational clinical study and *in vitro* assay

Patients and sampling. Adult patients undergoing living donor kidney transplantation (LD-KTx) at University Hospital were invited to participate after written informed consent (approved by Regional Ethical Review Board in Stockholm).[35] CKD was classified as abnormalities of kidney function that persists for > 3 months, i.e., low glomerular filtration rate (GFR) or elevated urine albumin should be detectable for at least 90 days, while kidney failure referred to patients with a GFR < 15 ml/min/1.73m^2 that require renal replacement therapy. For all patients, epigastric arteries and blood were obtained at the time of LD-KTx and stored for analysis. In short, epigastric arteries from 16 kidney failure patients were scored for calcification and fibrosis. Calcification, analysed by a trained pathologist on tissue sections (1-2 µm thick), was stained with von Kossa (silver nitrate plus nuclear fast red). The degree of medial calcification was graded from 0 (no calcification) to 3 (severely calcified). Patients with severely calcified (n=8) and non-calcified (n=8) vessels were matched for age, sex, CVD, statin usage, and presence of diabetes. Data was also obtained for % calcification in

epigastric artery vessel segments, measured semi-quantitatively in a previous study.[33,34] Due to the specific aims of the previous study and patients included in the present study, data from only 10/16 kidney failure patients' vessel segments were obtained. High sensitivity C-reactive protein (hsCRP) and creatinine quantification were performed by routine laboratory tests. Serum 8-OHdG was determined by competitive enzyme-linked immunosorbent assay (ELISA) using High Sensitivity 8-Hydroxydeoxyguanosine (8-OHdG) ELISA Assay Kit (JaICA). Patient epigastric artery segments were used for subsequent protein and RNA expression analysis, and uremic serum from patients with calcified vessels was used for *in vitro* calcification experiments.

In vitro calcification assay. Pooled uremic serum was obtained from the 8 patients presenting severe epigastric calcification (Table 1). Non-CKD controls serum (n=8) from the PRIMA-control cohort consisted of an age- and sex-matched population randomly selected from the Stockholm Region, Sweden, by the Statistics Bureau of Sweden—a government agency.[35] Human aortic VSMCs (ATCC) from a healthy male donor were incubated between passages 4-7 with one of the following conditions: control (DMEM), high phosphate (2.5mmol/L sodium phosphate), non-CKD control serum (2.5mmol/L sodium phosphate + 10% pooled non-CKD control serum), or uremic serum (2.5mmol/L sodium phosphate + 10% pooled uremic serum). Cells were incubated for 7 days (37°C, 5% CO_2). On day 7, cells were harvested for calcium content assay (IRDye® 800CW BoneTag™, Odyssey® CLx Infrared Imaging System, LI-COR) and qPCR analysis, or fixed for senescence-associated β-galactosidase (SA-β-Gal) staining.

mRNA expression. For human epigastric arteries and cultured VSMCs, total RNA extraction was performed using RNeasy kit (Qiagen) and RNA analysed using NP80 Nanophotometer (Implen). Maxima First Strand cDNA Synthesis Kit (ThermoFisher Scientfic) was used and qPCR was performed using PerfeCTa SYBR® Green SuperMix Low ROX (Quantabio). Data were analysed using QuantStudio 7 Flex Real-Time PCR System (Applied Biosystems) and relative expression was calculated according to the 2ΔΔCt method, using GAPDH as reference gene. Primer sequences used are detailed in Supplementary Table S2. mRNA expression in the form of Ct value (housekeeping gene) / Ct value (gene of interest) was also calculated to correlate with other genes of interest, % calcification of vessels, hsCRP, and osteogenic genes.

Immunofluorescence. 4 µm-sections of human epigastric arteries were incubated with primary antibodies: anti-p16 (ab189034, Abcam), anti-p21 (SAB1306168, Sigma), anti-NRF2 (SAB4501984, Sigma), and an appropriate secondary antibody (anti-rabbit IgG, SAB4600407, Sigma). Fluorescence was observed with Axio Observer Z1 motorized inverted fluorescence microscope (Zeiss).

Statistics. Statistical analysis was performed using GraphPad Prism v8.0.2 (San Diego, CA, USA). For categorical variables, Chi-squared test was used to compare groups. For all continuous variables, Kolmogorov–Smirnov test was used to test for normality. Afterwards, group comparisons were performed using independent t-test for normally distributed data or Mann–Whitney test for non-parametric data. Comparisons between three or more groups were performed using one-way ANOVA followed by Tukey's post hoc tests. Pearson or Spearman correlations between two quantitative variables were performed depending on data distribution. P-values <0.05 were considered statistically significant.
